# The Role of BMI Group on the Impact of Weight Bias Versus Body Positivity Terminology on Behavioral Intentions and Beliefs: An Experimental Study

**DOI:** 10.3389/fpsyg.2019.00634

**Published:** 2019-03-22

**Authors:** Sarah-Jane F. Stewart, Jane Ogden

**Affiliations:** School of Psychology, University of Surrey, Guildford, United Kingdom

**Keywords:** obesity, weight bias, body positivity, experiment, beliefs, causality, behavior

## Abstract

This experimental study investigated the role of BMI on the impact of weight bias vs body positivity terminology on behavioral intentions and beliefs about obesity. Participants (*n* = 332) were randomly allocated to two conditions to receive a vignette depicting an image of a person with obesity using either weight bias (*n* = 164) or body positivity (*n* = 168) terminology. Participants were divided into three groups based upon their BMI category (normal weight *n* = 173; overweight *n* = 92; obese *n* = 64). They then completed measures of behavioral intentions, obesity illness beliefs, and fat phobia. Although there were several differences in beliefs by BMI group, the results showed no differences between weight bias or body positivity terminology on any measures. There were, however, significant BMI group by condition interactions for beliefs about obesity relating to personal control and treatment control. *Post hoc* tests showed that weight bias resulted in reduced personal control in the obese BMI group compared to other participants. Weight bias also resulted in higher personal control over obesity in normal weight individuals compared to body positivity. People with obesity reported higher treatment control when exposed to weight bias compared to overweight participants, whereas normal weight participants reported greater treatment control when exposed to body positivity compared to both other groups. To conclude, the impact of weight bias and body positivity information is not universal and varies according to the BMI of the audience and the outcome being measured; whereas people of normal weight may benefit from weight bias there is no evidence that obese people benefit from body positivity. Implications for the prevention and treatment of obesity are discussed.

## Introduction

Weight bias describes negative attitudes toward those who are perceived to have surplus body weight (Pearl and Puhl, 2018) and has been described within the literatures focusing on weight stigma, discrimination, and prejudice (Puhl and Suh, 2015). These attitudes are embedded in negative stereotypes inclusive of both characterological blame (persons with overweight and obesity are lazy, sloppy, incompetent, and lack willpower) and behavioral blame (that they do not exercise enough and have excessive dietary consumption) (Puhl and Brownell, 2001). Research has explored the potential negative consequences of weight bias. The literature often uses terms such as weight bias, weight stigma, and weight discrimination interchangeably. For the purposes of this study, the term weight bias is used throughout.

In terms of discrimination, research suggests that weight bias can induce overt forms of discrimination in a variety of settings inclusive of healthcare, education, and employment, (Puhl and Heuer, 2010). Research also indicates links between weight bias and body dissatisfaction. For example, Jackson et al. (2015) found that perceived weight discrimination was related to higher levels of depressive symptoms and lower quality of life and life satisfaction. Furthermore, some research also suggests a negative impact of eating behavior and weight gain. For example, the cyclic obesity weight-based stigma (COBWEBS) states that being victim to weight bias causes an increase in stress and a cascade of emotional, physiological, and behavioral responses which promote increased dietary consumption and weight gain (Tomiyama, 2014). This is supported by a wealth of research illustrating a link between weight bias and maladaptive eating behaviors (King et al., 2013; Puhl and Suh, 2015; O’Brien et al., 2016; Vartanian and Porter, 2016; Araiza and Wellman, 2017). Some research has also addressed the impact of weight bias internalization (WBI) which refers to the self-directed negative and shaming weight-related stereotypes and attitudes toward oneself (O’Brien et al., 2016). For example, Zuba and Warschburger (2017) found that WBI mediated the relationship between BMI, emotional functioning, and eating behavior in children aged between 7 and 11. Some research has also addressed the impact of WBI on physical activity although the findings are less consistent (Schvey et al., 2013; Pearl and Puhl, 2018).

Research therefore indicates that weight bias is common and can have many detrimental effects. Some recent research, however, indicates some potential benefits to weight bias, particularly in terms of promoting healthy behaviors. For example, Puhl et al. (2018) showed that those who experienced more stigma and had greater WBI were more likely to report attempting to lose weight in the past 12 months and dieting. Likewise, Koball et al. (2018) found higher motivation to attempt to lose weight in those who experienced higher WBI. Similarly, weight bias may also be a deterrent to weight gain. In line with the social contagion model of obesity whereby obesity increases through social norms (Christakis and Fowler, 2007), experimental research by Robinson and Kirkham (2014) indicated that increased exposure to obesity leads to an increased likelihood of judging overweight people to be “healthy” weights and the normalization of heavier weights. Similarly, experimental research by Boothroyd et al. (2012) showed that participants who repeatedly viewed large body sizes to be desirable subsequently reported decreased liking and preference for thinner body sizes. Further, qualitative data indicates that moments of weight bias or stigma can sometimes act as the teachable moment people need to change their behavior and lose weight (Ogden and Clementi, 2010).

The notion of weight bias has therefore generated a wealth of research, mostly focusing on its potential impact. In parallel, the body positivity movement has gained momentum over recent years emphasizing celebrating one’s body regardless of its size and shape (Marcus, 2016). As such, body positivity can be considered the conceptual opposite to weight bias (Puhl and Brownell, 2001). In line with this, research demonstrating the detrimental impact of weight bias triggered the “health at every size” (HAES) movement, aimed to promote well-being through the eradication of weight bias and promotion of body positivity (Bacon, 2010; Tomiyama, 2014; Penney and Kirk, 2015). This movement has campaigned to re-educate health professionals and change the way obesity is managed by shifting the emphasis away from models of obesity which lead to blame and, it is argued, exacerbate the obesity problem (Bacon and Aphramor, 2011). Further, both the notion of body positivity and HAES reflect the ideals of the fat-acceptance movement (Marcus, 2016). While less research has explored the impact of body positivity, some studies tentatively point to both potential benefits and costs.

In terms of the benefits of body positivity, Tylka and Wood-Barcalow (2015) conducted a review of the evidence and concluded that there was evidence of the link between positive body image and increased psychological well-being. Likewise, Frederick et al. (2016) found that higher body satisfaction was correlated with higher self-esteem and life satisfaction in their large scale online cross-sectional survey. Similarly, Gillen (2015) reported a correlation between higher body satisfaction, an increase in self-care behaviors and lower depression symptomology, and qualitative research by Wood-Barcalow et al. (2010) reported that those women who expressed love and acceptance for their bodies also engaged in more self-care behaviors such as moderate exercise and intuitive eating.

Some research, however, suggests that body positivity may also have negative consequences. For example, Heinberg et al. (2001) argued that positive body image and higher levels of body acceptance may lead to a decreased motivation to engage in healthy behaviors and a number of cross-sectional studies indicate that positive body image is associated with a decrease in weight loss behaviors (Carroll et al., 2007; Andrew et al., 2016). Furthermore, it has been argued that a focus on body positivity and body confidence, rather than body size, may lead to weight normalization in parallel with experimental on exposure to normalized body sizes (Robinson and Kirkham, 2014; Robinson, 2017). In line with this, a survey of over 23,000 British people indicated an increasing underestimation of body weight which was associated with the normalization of obesity (Muttarak, 2018). Furthermore, those under estimating their body weight were not only less likely to attempt to lose weight but also overestimated their health status. Accordingly, body positivity (regardless of body weight) could decrease motivation to undertake health behaviors, promote the normalization of heavier weights, and in turn encourage people to overestimate their health status.

Research therefore suggests that weight bias is on the increase which has resulted in the call for body positivity and a focus on HAES. Research also indicates that weight bias can have negative consequences whereas body positivity may promote psychological well-being. Research exploring both these areas, however, is sometimes contradictory. There are several possible explanations for this. First, many studies in this area rely upon cross-sectional or qualitative designs which limits conclusions about causality (see Pearl and Puhl, 2018 for a review). Therefore, rather than the perception of weight bias causing eating pathology or depression, the reverse may well be the case. Second, different research studies include different populations often varying in body weight. For example, much research exploring the impact of body positivity involves participants of normal weight yet generalizes to those who are either overweight or obese. In contrast, research exploring the impact of weight bias involves participants who are overweight or obese yet generalizes to those who are of normal weight. It is therefore assumed that findings are consistent across all body weights, yet this has not been directly tested which has implications for the prevention of obesity (i.e., in those of normal weight) or its treatment (i.e., those who are already overweight). Furthermore, while weight bias and body positivity may impact upon behavior and subsequent weight the mechanisms of this process remain unclear. Finally, while these two literatures exist exploring either the impact of weight bias or the impact of positivity, the relationship between these two approaches has yet to be explored.

This study therefore combined these two literatures using an experimental design to evaluate the impact of both weight bias and body positivity terminology on participants’ health outcomes. The study also explored whether this impact varied according to the weight status of the participant as a means to model the differential effects for prevention and treatment. In addition, the study explored the impact of both weight bias and body positivity on beliefs about obesity as a means to explore the role of beliefs as potential mechanisms. In particular, the study focused on beliefs about obesity with a focus on perceptions of control and the causes and consequences of obesity and whether either weight bias or body positivity promoted a focus on behavioral, medical, or social factors. Measures of aspects of weight bias, stigma, or weight discrimination often include items relating to the role of behavior and psychological factors as causes for obesity (DePierre and Puhl, 2012). From this perspective, endorsing a behavioral or psychological model of obesity is considered core to the notion of weight bias. In line with this, those campaigning for the eradication of weight bias are largely in support of a medical model of obesity as a means to avoid issues of failed control and blame (Pearl and Lebowitz, 2014). The present study therefore explored the impact of both weight bias and body positivity terminology on beliefs about obesity as a means to explore whether these approaches changed participants’ perceptions of the controllability of obesity and whether it was a product of behavior or less controllable factors such as biology.

## Materials and Methods

### Design

This study used an experimental design with two between subject variables: condition (weight bias terminology vs body positivity terminology) and BMI group (normal weight vs overweight vs obese). Participants were randomly allocated to one of the two conditions and received information describing a person with obesity using either weight bias or body positivity terminology. BMI groups were determined *post hoc*. Dependent variables were behavioral intentions, beliefs about obesity, and fat phobia.

### Participants

Participants were recruited via opportunity and snowballing methods using social media. Eligibility criteria were: aged between 17–75, with a BMI of at least 18.5. A total of 401 participants started the online questionnaire; 13 were removed because they were underweight (BMI < 18.5); 45 participants had had weight loss surgery and were therefore also excluded from the analyses due the unique beliefs demonstrated by this population and 11 did not complete all measures. The final sample consisted on 332 participants: weight bias (*n* = 164) or body positivity (*n* = 168). Participants were grouped by BMI as follows: normal weight: BMI 18.5–24.9 (*n* = 173); overweight: BMI 25–29.5 (*n* = 92); obese BMI ≥ 30 (*n* = 67).

### The Interventions

The two conditions involved exposure to an image of a person with obesity described using either weight bias or body positivity terminology. Exposure lasted for 3 min. The images were of a man or a woman with obesity and were used with permission from Oldham and Robinson (2016). Both images were Caucasian, aged 18–30 with BMI ≥ 30. The photographs had a black square obscuring the view of the model’s face. Both photographs were full length and depicted the individual with their arms by their sides wearing regular fitting short sleeved t-shirts and full-length trousers, in order to stay as naturalistic to how the public are exposed to obese people in every-day life. Participants received a gender matched image. These images were accompanied by short vignettes using either weight bias or body positivity terminology as follows.

#### Weight Bias

The vignettes were developed to include aspects of weight bias, particularly characterological blame and were formed on the basis of research by Puhl and Brownell (2001) exploring the most common anti-fat beliefs. The vignettes stated: “*This is Peter/Susan. He/she is not very intelligent and is not very ambitious, particularly in terms of his/her work. He/she does not have many friends and is not very liked by his/her colleagues. He/she has often thought about trying to lose some weight, but is very lazy, undisciplined and cannot be bothered because it is too much effort.*”

#### Body Positivity

These vignettes were inspired by the terminology and concerns expressed by the body positivity movement within online communities, inclusive of body confidence, self-respect, and feeling beautiful irrespective of body size (Marcus, 2016). The vignettes stated: “*This is Peter/Susan. He/she loves to challenge himself/herself both in his/her personal life and at work, and always strives to achieve. He/she knows he/she isn’t perfect, but still feels confident in his/her own body and respects himself/herself. He/she does not feel that he/she should change how he/she looks to fit in with everybody else. He/she knows that he/she should not be embarrassed by his/her body-size and believes that he/she is beautiful.*”

Participants were asked to read the vignettes and then complete the following measures.

### Measures

All participants completed the following measures after the interventions. Cronbach’s alpha was used to assess reliability where appropriate.

#### Behavioral Intentions

Behavioral intentions were measured using a nine-item scale (Ogden and Arulgnanaseelan, 2017). Participants rated the extent to which they intend to engage in a series of behaviors on a Likert-scale ranging from 1 (not at all) to 5 (totally) on measures of: *intentions to eat healthily* (three items: e.g., “intend to eat more healthily”; alpha = 0.8); *intentions to exercise* (three items: e.g., “intend to be more active”; alpha = 0.8); *intentions to manage their weight* (three items: e.g., “intend to lose weight”; alpha = 0.9).

#### Beliefs About Obesity

Participants completed measured relating to beliefs about the causes and consequences of obesity and their illness perceptions.

##### Beliefs about the causes of obesity

Participants rated the extent to which they believe a series of items were causes of obesity using a 12-item scale, rated from not at all (1) to totally (5) (Ogden et al., 2001). The scale included the following causes: *medical* (three items: e.g., “genetics/inheritance”; alpha = 0.6); *psychological* (three items: “low self-esteem,” “anxiety/stress,” “depression”; alpha = 0.7); *eating behavior* (three items: e.g., “eating too much”; alpha = 0.55); *lack of exercise* (three items: e.g., “not enough exercise”; alpha = 0.8); *environmental* (three items: e.g., “an increasing number of takeaway restaurants”; alpha = 0.7).

##### Beliefs about the consequences of obesity

Participants rated the extent to which they believe a series of items were consequences of obesity using a nine-item scale, rated from not at all (1) to totally (5) (Ogden et al., 2001). The scale included the following consequences: *medical* (three items, e.g., “high blood pressure”; alpha = 0.7); *psychological* (three items, e.g., “depression/anxiety”; alpha = 0.8); *social* (three items, e.g., “difficulty getting to work”; alpha = 0.6).

##### Illness perceptions

Participants completed subscales of the Illness Perception Questionnaire (IPQ-R, Moss-Morris et al., 2002) which were of most relevance to the controllability and consequences of obesity: *consequences* (three items: e.g., “weight problems strongly affect the way others see you”; alpha = 0.7); *treatment control* (three items: e.g., “treatment can control weight problems”; alpha = 0.7); *personal control* (three items: e.g., “you can have the power to affect your own weight problem”; alpha = 0.6); *emotions* (three items: e.g., “when you think about weight problems, you get upset”; alpha = 0.8); *meaning* (three items: e.g., “you understand your weight problem”; alpha = 0.6). Items were rated from “strongly disagree” (1) to “strongly agree” (5).

#### Fat Phobia

Participants completed the “Fat Phobia Scale – Short Form” (Bacon, Scheltema and Robinson, 2001; alpha = 0.9) as a measure of weight bias.

#### Demographics

Participants described their age, gender, weight, height, and whether they had had weight loss surgery.

### Procedure

Favorable ethical approval was obtained from the University ethics committee. The questionnaire was completed online. After providing consent, participants provided their demographic information and were then randomly allocated to one of the two conditions (body positivity vs weight bias) to view a gender-matched photo of a person with obesity (BMI ≥ 30) and read a short vignette reflecting the condition. To encourage participants to focus on the image and vignette they were told that they would be asked a few questions about the stimuli (their name, and some adjectives used to describe them). Participants then completed measures of behavioral intentions and beliefs about obesity.

## Results

### Participant Demographics

Demographics for all participants and by condition are shown in Table 1.

**Table 1:** Participant demographics by condition.

Variable	All (*n* = 332)	Body positive (*n* = 168)	Weight bias (*n* = 164)	*t*-Test values/χ^2^ values
Gender	Male = 69	Male = 33	Male = 36	χ^2^(1) = 0.27,
	Female = 263	Female = 135	Female = 128	*p* = 0.60
Age	*M* = 38.8	M = 38.9	*M* = 38.7	*t*(375) = 0.77,
	*SD* = 15.6	SD = 14.5	*SD* = 15.6	*p* = 0.44
	Range = 17–75	Range = 19–75	Range = 17–75	
Ethnicity	White = 311	White = 159	White = 152	χ^2^(2) = 0.60,
	Black = 0	Black = 0	Black = 0	*p* = 0.74
	Asian = 10	Asian = 4	Asian = 6	
	Other = 11	Other = 5	Other = 6	
BMI	*M* = 26.8	*M* = 28.3	M = 27.4	*t*(375) = 1.07,
	*SD* = 7.7	*SD* = 8.3	SD = 8.6	*p* = 0.29
	Range = 18.5–61.9	Range = 18.8–60.5	Range = 18.5–61.9	

The majority of participants were female, White with a mean age of 38 years. The mean BMI was 26.8 ranging from 18.5 to 61.9. There were no significant differences in demographics by condition indicating that the randomization of participants across the two conditions was successful. Due to the potential differences in the impact of weight bias and body positivity terminology on men and women, gender was used as a covariate in all subsequent analyses. The small sample of men meant that gender could not be used as an additional between subjects factor.

### Impact of Condition and BMI on Behavioral Intentions, Beliefs About Obesity, and Fat Phobia

The results were analyzed to explore the impact of condition and BMI group using a two-way ANCOVA with gender as the covariate.

#### Behavioral Intentions

Behavioral intentions by condition and BMI group are shown in Table 2.

**Table 2:** Impact of condition and BMI group on behavioral intentions.

	Weight bias (N = 164)			Body positivity (N = 168)			Main effect of condition			Main effect of BMI			Condition X BMI		
	Normal (*N* = 92) *M*(*SD*)	O/W (*N* = 42) *M*(*SD*)	Obese (*N* = 30) *M*(*SD*)	Normal (*N* = 81) *M*(*SD*)	O/W (*N* = 50) *M*(*SD*)	Obese (*N* = 37) *M*(*SD*)	*F*	*P*	η*_p_*^2^	*F*	*p*	η*_p_*^2^	*F*	*p*	η*_p_*^2^
Eating intentions	4.39 (0.55)	4.40 (0.66)	4.22 (0.43)	4.44 (0.51)	4.39 (0.63)	4.26 (0.57)	0.67	0.41	0.00	0.75	0.47	0.01	0.98	0.38	0.01
Exercise intentions	4.17 (0.68)	4.03 (0.84)	3.72 (1.00)	4.23 (0.70)	4.22 (0.71)	3.84 (0.91)	1.90	0.17	0.01	8.40	<0.001	0.05	0.06	0.94	0.00
Weight management intentions	4.48 (0.62)	4.33 (0.67)	4.21 (0.67)	4.51 (0.52)	2.27 (0.71)	4.07 (0.79)	0.25	0.44	0.00	7.94	<0.001	0.05	0.44	0.64	0.00

The results showed no main effect of condition for behavioral intentions. However, the results showed a significant main effect of BMI group for exercise and weight management intentions but not for intentions relating to eating behavior. The means indicate that regardless of condition, those in the obese BMI group reported lower intentions to exercise and manage their weight than those in the normal (ps < 0.05) and overweight (ps < 0.05) BMI groups. The results also showed no condition by BMI interactions. This indicates that participants showed no difference in their responses to either the weight bias or body positivity vignette in their behavioral intentions and that this response was unrelated to their BMI group.

#### Beliefs About Obesity

The results for beliefs about obesity are shown in Tables 3, 4.

**Table 3:** Impact of condition and BMI on beliefs about the causes and consequences of obesity.

	Weight bias (*N* = 164)			Body positivity (*N* = 168)			Main effect of condition			Main effect of BMI			Condition X BMI		
	Normal *N* = 92 *M*(*SD*)	O/W *N* = 42 *M*(*SD*)	Obese *N* = 30 *M*(*SD*)	Normal *N* = 81 *M*(*SD*)	O/W *N* = 50 *M*(*SD*)	Obese *N* = 37 *M*(*SD*)	*F*	*p*	η*_p_*^2^	*F*	*p*	η*_p_*^2^	*F*	*p*	η*_p_*^2^
Psychological causes	3.78 (0.56)	3.79 (0.59)	4.01 (0.45)	3.79 (0.48)	3.78 (0.57)	4.00 (0.66)	0.39	0.53	0.00	4.62	0.01	0.03	0.14	0.87	0.00
Medical causes	3.60 (0.65)	3.52 (0.54)	3.60 (0.74)	3.45 (0.70)	3.45 (0.83)	3.75 (0.62)	1.36	0.24	0.00	3.17	0.04	0.02	1.89	0.15	0.01
Environmental causes	3.59 (0.74)	3.87 (0.60)	3.71 (0.87)	3.59 (0.85)	3.59 (0.88)	3.66 (0.80)	1.54	0.22	0.01	0.99	0.37	0.01	1.20	0.30	0.01
Exercise causes	4.22 (0.55)	4.29 (0.41)	4.36 (0.49)	4.36 (0.55)	4.25 (0.49)	4.36 (0.45)	0.24	0.62	0.00	0.51	0.60	0.00	1.16	0.31	0.01
Eating causes	4.18 (0.54)	4.21 (0.47)	4.38 (0.49)	4.30 (0.48)	4.25 (0.48)	4.23 (0.54)	0.01	0.94	0.00	0.38	0.68	0.00	1.84	0.16	0.01
Medical consequences	4.36 (0.45)	4.24 (0.44)	4.27 (0.43)	4.34 (0.41)	4.27 (0.53)	4.28 (0.65)	0.01	0.95	0.00	1.56	0.21	0.01	0.03	0.97	0.00
Psychological consequences	3.96 (0.54)	4.01 (0.47)	4.21 (0.60)	3.96 (0.56)	4.04 (0.55)	4.12 (0.65)	0.12	0.73	0.00	3.15	0.04	0.02	0.30	0.74	0.00
Social consequences	3.22 (0.76)	3.19 (0.71)	3.80 (0.68)	3.26 (0.81)	3.35 (0.54)	3.45 (0.79)	0.17	0.69	0.00	8.29	<0.001	0.05	2.65	0.07	0.02

**Table 4:** Impact of condition and BMI on beliefs about obesity (IPQ) and fat phobia.

	Weight bias (*N* = 164)			Body positivity (*N* = 168)			Main effect of condition			Main effect of BMI			Condition X BMI		
	Normal (*N* = 92) *M*(*SD*)	O/W (*N* = 42) *M*(*SD*)	Obese (*N* = 30) *M*(*SD*)	Normal (*N* = 81) *M*(*SD*)	O/W (*N* = 50) *M*(*SD*)	Obese (*N* = 37) *M*(*SD*)	*F*	*p*	η*_p_*^2^	*F*	*p*	η*_p_*^2^	*F*	*p*	η*_p_*^2^
Beliefs about consequences	4.00 (0.73)	4.05 (0.60)	4.48 (0.46)	4.11 (0.57)	4.11 (0.51)	4.17 (0.71)	0.31	0.58	0.00	4.83	0.01	0.03	2.78	0.06	0.02
Beliefs about personal control	4.51 (0.53)	4.43 (0.66)	3.90 (0.77)	4.30 (0.67)	4.29 (0.62)	4.12 (0.70)	0.23	0.63	0.00	8.42	<0.001	0.05	3.02	0.05	0.02
Beliefs about treatment control	3.22 (0.77)	3.02 (0.70)	3.40 (0.72)	3.44 (0.66)	2.99 (0.78)	3.05 (0.91)	0.27	0.61	0.00	5.76	0.001	0.03	3.30	0.04	0.02
Beliefs about emotions	4.01 (0.61)	3.82 (0.77)	4.34 (0.66)	3.93 (0.65)	4.02 (0.50)	4.05 (0.89)	0.75	0.39	0.00	3.10	0.05	0.02	2.69	0.07	0.02
Beliefs about meaning	4.19 (0.71)	4.08 (0.98)	3.55 (0.92)	4.30 (0.68)	3.96 (0.77)	3.78 (0.87)	0.61	0.44	0.00	12.56	<0.001	0.07	1.05	0.35	0.01
Fat phobia	3.44 (0.65)	3.60 (0.55)	3.67 (0.71)	3.56 (0.69)	3.58 (0.61)	3.65 (0.67)	0.14	0.71	0.00	1.83	0.16	0.01	0.40	0.67	0.00

##### Beliefs about the causes and consequences of obesity (see Table 3)

The results showed no main effect of condition for beliefs about the causes or consequences of obesity. However, the results showed a main effect of BMI group for some measures of beliefs with those in the obese BMI group reported stronger beliefs about psychological causes, medical causes, psychological consequences, and social consequences (ps < 0.05). No differences by BMI group were found for beliefs about environmental, exercise or eating causes, or medical consequences. Further, no condition by BMI group interactions was found for any beliefs about causes or consequences although the condition by BMI group interaction for social consequences approached significance. This indicated that participants showed no differences in their responses to either the weight bias or body positivity vignette and that these responses were unrelated to their BMI group.

##### Illness perceptions (see Table 4)

The results showed no main effect of condition on illness perceptions. There were significant main effects of BMI group for beliefs about obesity in terms of general consequences, personal control, treatment control, emotions, and meaning. The means indicated that those in the obese BMI group reported greater beliefs about the consequences and emotional impact and lower beliefs about the meaning of obesity and personal control than those who were in either the overweight (ps < 0.05) or normal weight (ps < 0.05) BMI groups. Those in the obese BMI group also reported lower beliefs relating to treatment control compared with those who were normal weight, but stronger beliefs relating to treatment control compared with those who were overweight (ps < 0.05). The results showed no significant condition by BMI group interactions for beliefs about consequences, emotions, or meaning although the interactions for beliefs about consequences and emotions approached significance. Significant interactions, however, were found for beliefs about personal control and treatment control which were explored using *post hoc* tests (LSD).

For beliefs about personal control, *post hoc* tests showed that whereas BMI group had no impact on how participants responded to the body positivity vignette, BMI did influence how participants responded to the weight bias vignette. In particular, obese participants reported lower perceptions of personal control over obesity after viewing the weight bias information compared to both the normal weight participants [*t*(39.54) = 3.88, *p* < 0.001, *d* = 0.87] and the overweight participants [*t*(71) = 3.02, *p* = 0.004, *d* = 0.52]. In contrast, the normal weight participants showed a similar response to the weight bias information as the overweight participants [*t*(136) = 0.68, *p* = 0.50]. Furthermore, those with a normal BMI reported greater personal control over obesity after viewing the weight bias information compared to the body positivity information [*t*(176) = −2.36, *p* = 0.02, *d* = 0.35].

For beliefs about treatment control, *post hoc* tests showed that exposure to body positivity resulted in higher perceptions of treatment control over obesity in normal weight participants compared to both overweight [*t*(130) = 3.53, *p* = 0.001, *d* = 0.62], and obese participants [*t*(53.27) = 2.31, *p* = 0.03, *d* = 0.48]. In contrast, exposure to weight bias resulted in a greater perceptions of treatment control over obesity for obese participants compared to overweight participants [*t*(71) = −2.17, *p* = 0.03, *d* = 0.51]. In addition, normal weight participants reported greater perceptions of treatment control after the body positivity condition compared to those exposed to weight bias [*t*(176) = 2.14, *p* = 0.03, *d* = 0.32].

#### Fat Phobia

The impact of condition and BMI on fat phobia is shown in Table 4. The results showed no main effect of condition or BMI nor a condition by BMI interaction for fat phobia.

## Discussion

The present study aimed to explore the impact of weight bias and body positivity terminology on participants’ behaviors and beliefs about obesity using an experimental approach and to assess whether the impact of these two terminologies varied by BMI group. The results showed no main effect of condition for any measures indicating no differences in participants’ responses to terminology reflecting either weight bias or body positivity. This conflicts with much correlational and qualitative research which has suggested a detrimental effect of weight bias compared to a beneficial effect of body positivity (see Tylka and Wood-Barcalow, 2015; Pearl and Puhl, 2018 for reviews). However, it also conflicts with studies which suggest the reverse and have argued that weight bias may have benefits with body positivity doing harm (e.g., Koball et al., 2018; Puhl et al., 2018). This may be due to several factors. First, it could reflect the correlational nature of previous research with previous findings indicating association rather than causation. In line with this, rather than weight bias having a detrimental impact upon health outcomes, those who experience negative states may perceive that they have also experienced more weight bias. Likewise, those with more positive health outcomes may also experience body positivity without there being a causal relationship. Accordingly, the impact of weight bias and body positivity may not be as robust as sometimes suggested. Second, this may reflect the role of individual differences, particularly the impact of body weight.

In support of this second explanation, the results found that the impact of the intervention was dependent upon BMI group for beliefs relating to both personal control and treatment control. In particular, whereas participants who were obese reported lower perceptions of personal control over obesity after viewing the weight bias information compared to other participants, those with normal BMI reported greater personal control over obesity after viewing the weight bias information compared to the body positivity information. Therefore, weight bias seemed to reduce perceptions of personal control for those in the obese BMI group and increase perceptions of personal control for those who were of normal weight. The COBWEBS model (Tomiyama, 2014) predicts that those subjected to weight bias experience negative emotions which in turn can lead to pathological eating and weight gain which has found some support in the literature (see Pearl and Puhl, 2018 for a review). The results from the present study provide some support for these predictions for those in the obese BMI group who responded to weight bias terminology with poorer perceived personal control than other participants. This may reflect a sense of shame or blame and indicates that focusing on the negative characterological attributes of the obese target in the vignette reduced their sense of personal control. In contrast, however, the reverse was found for those in the normal weight BMI group who seemed to gain benefit from weight bias terminology and reported increased personal control. Accordingly, weight bias and body positivity may have an impact on some beliefs about obesity, particularly those relating to control, but this is dependent upon the body weight of the participant. Likewise, a similar pattern was also found for beliefs about treatment control. In particular, whereas weight bias resulted in greater perceptions of treatment control in those in the obese BMI group, those of normal weight responded to body positivity with greater perceptions of treatment control. Again, this indicates that the impact of either weight bias or body positivity depends of the weight of the individual. No interactions were found, however, for behavioral intentions or other measures of beliefs.

There are, however, some problems with this study that need to be considered. First, while using an experimental design enabled conclusions to be drawn about causality, this inevitably made the weight bias and body positivity interventions low on ecological validity. In a real-world setting outside of the laboratory, both these forms of terminology are persistent and ongoing and take many forms including images, text, and the spoken word. In contrast, the intervention used for the present study was short term and limited in its format which may well have minimized the impact of the different types of information presented. Future research is needed to develop ways to reflect more realistically the nature of both weight bias and body positivity while maintaining an experimental approach. Second, at the core of research exploring the impact of either weight bias or body positivity is actual behavior and weight change. The present study, however, only measured proxy variables in the form of behavioral intentions and beliefs. Future research is needed to assess these more objective outcomes. Third, research indicates that the impact of these approaches may be broad ranging emphasizing factors such as stress, blame, shame, depression, and body dissatisfaction. Research could also address these variables in future. Finally, the sample was opportunistic and therefore not necessarily representative and limits the generalizability of the findings. Given these limitations, however, the present study, does provide some preliminary experimental evidence for the impact of weight bias and body positivity on some health outcomes.

These results have some tentative implications for practice, particularly the treatment and prevention of obesity. In line with previous research (Tylka and Wood-Barcalow, 2015; Pearl and Puhl, 2018), the findings suggest that weight bias may have detrimental consequences and reduces perceptions of personal control in those who are obese while increasing their perceptions of treatment control. Accordingly, by focusing on negative characterological features of those who are obese, weight bias generates a sense of helplessness and encourages those who are themselves obese to look to external factors for help and support. From this perspective, weight bias may hinder obesity treatments which require behavior change and body positivity would seem to be a more productive way forward. In contrast, however, for those who are of normal weight, weight bias encouraged a greater sense of personal control whereas body positivity encouraged a move toward greater treatment control. Therefore, for those who remain of normal weight, and for whom prevention of obesity is key, weight bias may be a more useful approach.

## Conclusion

The present study aimed to directly explore the impact of both weight bias and body positivity terminology using an experimental design while accounting for BMI group. The results showed no differences overall between the effects of either weight bias or body positivity on either behavioral intentions or beliefs suggesting that the consequences of these approaches may not be as different, robust, or universal as sometimes predicted. The results, however, did show that at times, the impact of weight bias and body positivity were dependent on BMI. In particular, whereas weight bias decreased perceptions of personal control and increased perceptions of treatment control in those who were obese, those of normal weight responded with the reverse effect. Only limited benefits of body positivity were found. These results have some implications for practice and indicate that while weight bias may be detrimental for the treatment of obesity, it may be of more benefit for its prevention.

## Data Availability

Data are available on request.

## Ethics Statement

This study was carried out in accordance with the recommendations of University of Surrey Ethics Committee with written informed consent from all subjects. All subjects gave written informed consent in accordance with the Declaration of Helsinki. The protocol was approved by the University of Surrey Ethics Committee.

## Author Contributions

JO and S-JS designed the study. S-JS collected and analyzed the data and wrote the first draft. JO edited the manuscript. The study was completed as part assessment for the M.Sc. in Health psychology for S-JS supervised by JO.

## Conflict of Interest Statement

The authors declare that the research was conducted in the absence of any commercial or financial relationships that could be construed as a potential conflict of interest.
